# Supplementary material for: Neutralization of SARS‐CoV‐2 by highly potent, hyperthermostable, and mutation‐tolerant nanobodies
Source: EMBO J. 2021 Aug 9;40(19):e107985. doi: 10.15252/embj.2021107985 (PMC8420576; doi:10.15252/embj.2021107985)
Supplement: Supplementary file 1 — Appendix [file EMBJ-40-e107985-s002.pdf]

Appendix

Neutralization of SARS-CoV-2 by highly potent, hyperthermostable, and mutation-tolerant nanobodies

Thomas Güttler[1*], Metin Aksu[1*], Antje Dickmanns[2], Kim M. Stegmann[2], Kathrin Gregor[1], Renate Rees[1], Waltraud Taxer[1], Oleh Rymarenko[1], Jürgen Schünemann[1], Christian Dienemann[3], Philip Gunkel[1], Bianka Mussil[1], Jens Krull[1], Ulrike Teichmann[4], Uwe Groß[5], Volker C. Cordes[1], Matthias Dobbelstein[2]**, and Dirk Görlich[1]**

[1]Department of Cellular Logistics, [3]Department of Molecular Biology, [4]Animal facility, Max Planck Institute for Biophysical Chemistry, Am Fassberg 11, D-37077 Göttingen, Germany.

[2]Institute of Molecular Oncology, GZMB, University Medical Center, Justus-von-Liebig-Weg 11, D-37077 Göttingen, Germany.

[5]Institute of Medical Microbiology and Virology, University Medical Center, Kreuzbergring 57, D-37075 Göttingen.

* Equal contribution.

** Corresponding authors.

Content:

Appendix Figures S1 – S13

Appendix Tables S1 – S4

Appendix References

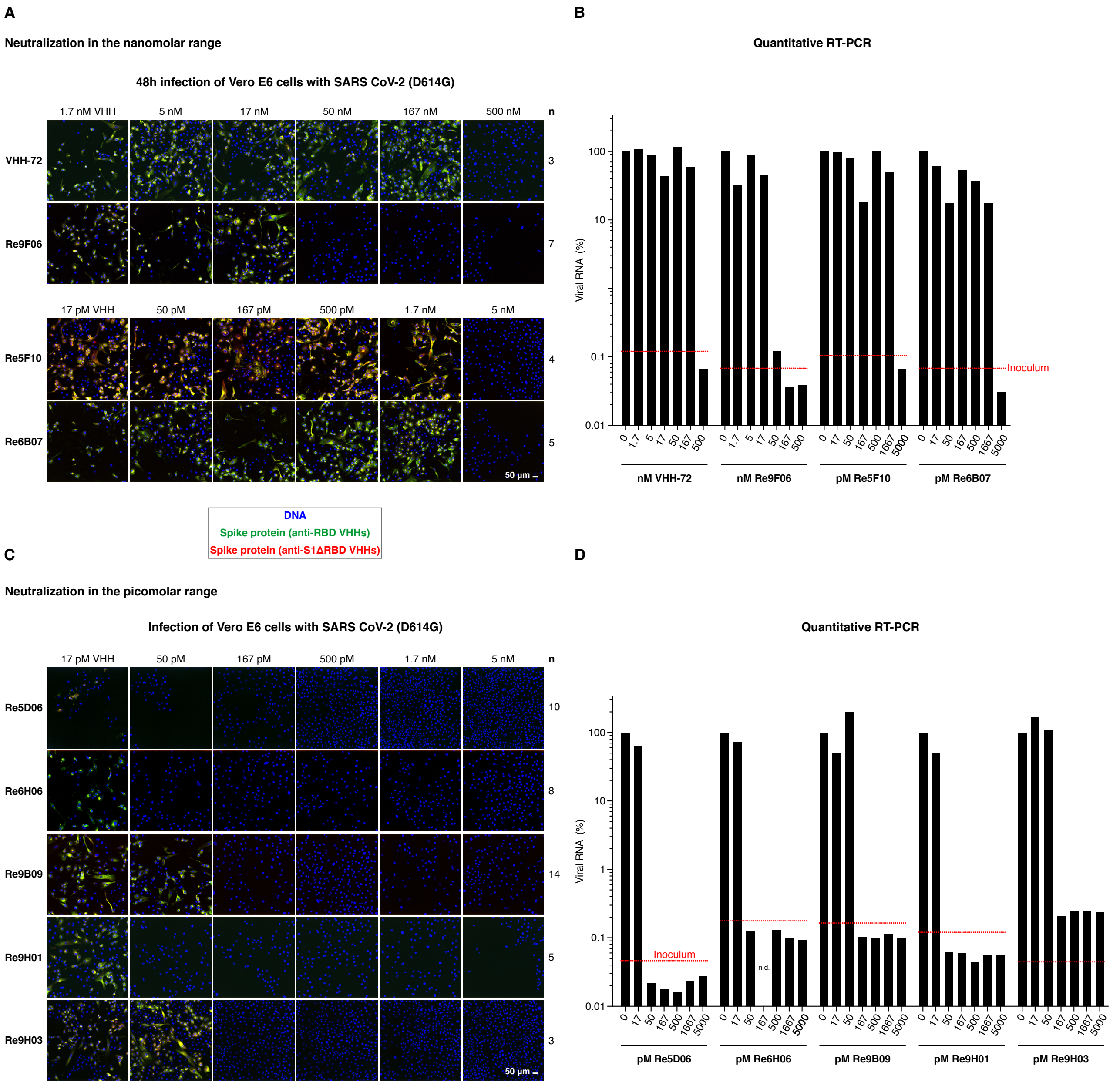

Figure S1 (related to Fig 3 and 4)

Neutralization of SARS-CoV-2 by VHH antibodies.

The figure panels show a biological replicate of the neutralization experiments presented in Fig 3 and 4. The indicated VHH antibodies (**A and B**: nanomolar neutralizers, **C and D**: picomolar neutralizers) were tested for their neutralization potency with the fluorescene-based readout (**A and C**) and by quantitative RT-PCR (**B and D**). See Fig 3A and B for more information on experimental details. "**n**" indicates the number of independent biological replicates.

For Re5D06, the cells were fixed 3 days after inoculation with SARS-CoV-2. Even after such a prolonged incubation, Re5D06 neutralized the virus potently at a concentration of 50 pM (IC99+). For all other nanobodies, cells were fixed 2 days after inoculation, as in Fig 3 and 4.

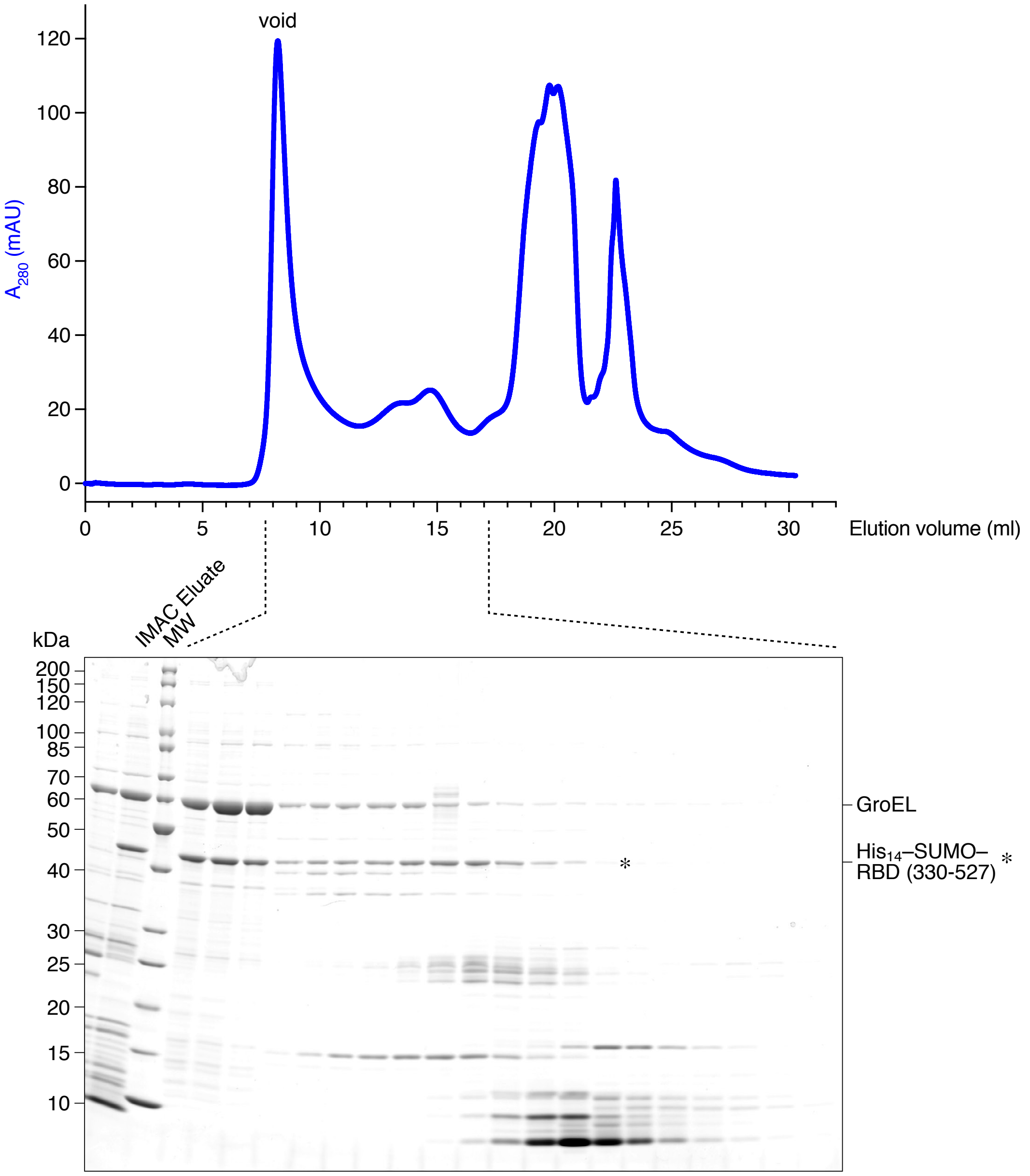

Figure S2 (related to Fig S3)

Expression and purification of the free RBD in the *E. coli* cytosol.

The SARS-CoV-2 receptor-binding domain (RBD, residues 330-527) was expressed in the cytosol of *E. coli* SHuffle® Express as an N-terminal fusion with a His_{14}-SUMO tag. Cells were lysed by sonication, the soluble lysate was obtained by ultracentrifugation and applied to a Ni-chelate column. Non-bound material was washed off with lysis buffer, and the fusion protein was eluted with buffer containing imidazole. The concentrated eluate was subjected to size exclusion chromatography (Superdex 200). The A_{280} elution profile is shown in blue; the indicated fractions were analyzed by SDS-PAGE and Coomassie staining. Note that the RBD is trapped by GroEL in the IMAC eluate and that the majority of the RBD elutes within the void volume of the column.

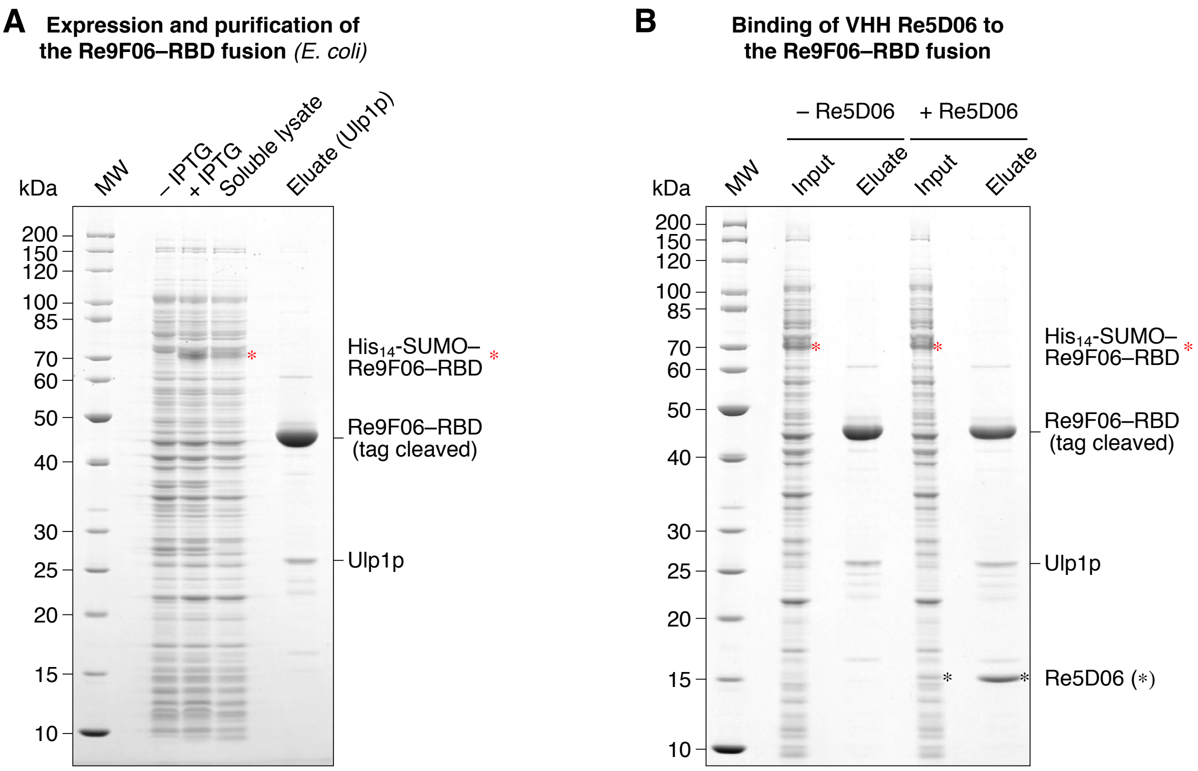

Figure S3 (related to Fig 6)
Soluble expression and purification of an Re9F06–RBD fusion.

(**A**) The SARS-CoV-2 RBD was expressed in the cytosol of *E. coli* SHuffle® Express (by induction with IPTG; "–/+ IPTG") as an Re9F06-RBD fusion with an N-terminal His_{14}-SUMO tag. Cells were lysed by sonication, the soluble lysate was obtained by ultracentrifugation and applied to a Ni-chelate column. Non-bound material was washed off with lysis buffer, and the Re9F06-RBD fusion was eluted by cleaving off the His_{14}-SUMO tag with the Ulp1p protease. The indicated fractions were analyzed by SDS-PAGE and Coomassie staining. Note the strong Re9F06-RBD fusion band in the eluted fraction. The yield was approximately 10 mg Re9F06-RBD fusion per liter of culture.

(**B**) Binding of the Re9F06-RBD fusion to Ni-chelate beads as in panel **A**, but in a small (analytical) scale. Where indicated, 1 μM untagged Re5D06 was added to the binding reaction; the beads were washed with lysis buffer containing 1 M NaCl, and elution was done as in **A** (with the Ulp1p protease). Co-elution of Re5D06 (marked with a black asterisk) with the Re9F06-RBD fusion indicates a specific RBD·Re5D06 interaction. The endogenous *E. coli* proteins present in the soluble starting lysate can be regarded as negative controls for the binding reaction.

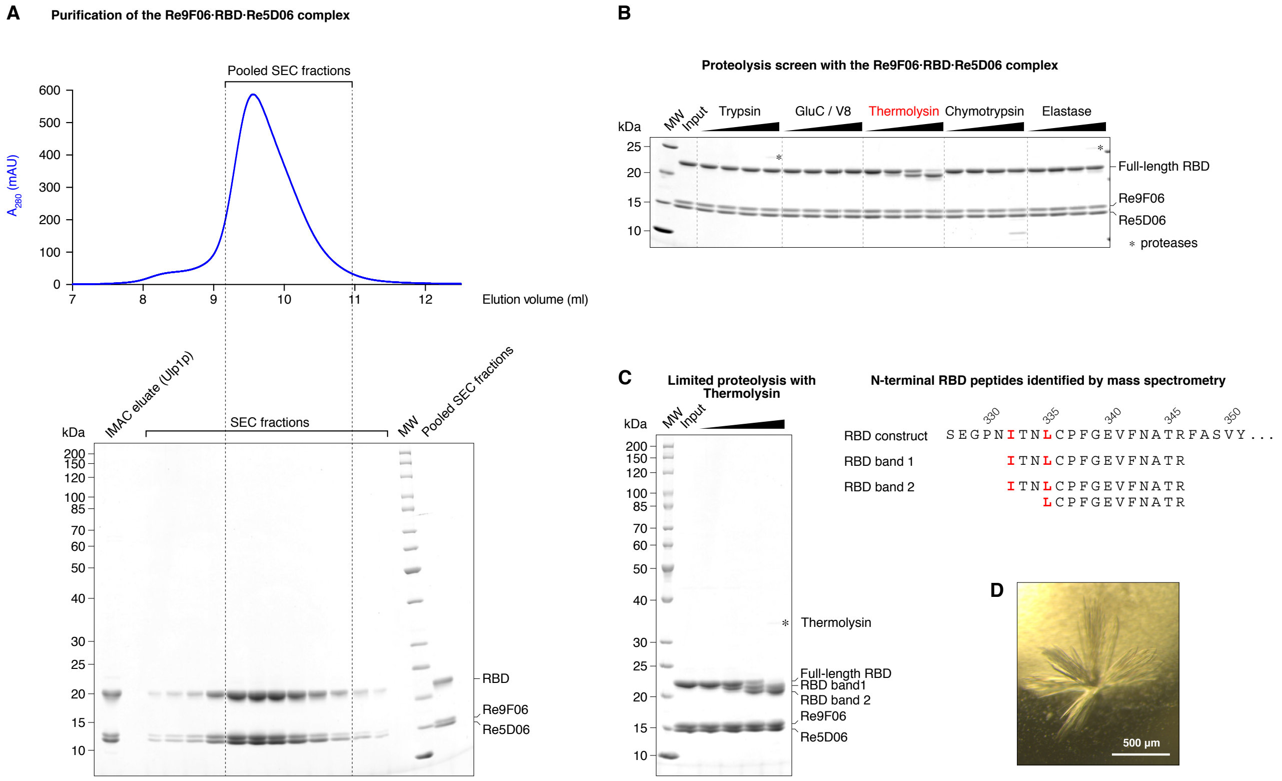

Figure S4 (related to Fig 6)

Purification and crystallization of the Re9F06·RBD·Re5D06 complex.

(**A**) The Re9F06·RBD·Re5D06 complex was expressed in *E. coli* and purified as described in "Materials and Methods". The last affinity chromatography step entailed protease (Ulp1p)-mediated elution of the complex from an IMAC column, and further purification was performed by gel filtration on a Superdex 75 column. The elution profile (A_{280}) was recorded **(upper panel)**, followed by analysis of the input material and eluate fractions by SDS-PAGE and Coomassie staining **(lower panel)**. Fractions that were pooled and further concentrated for crystallization screening are indicated.

(**B**) 4 µg of the Re9F06·RBD·Re5D06 complex were treated for 1 hour (at 23°C) with the indicated proteases (that vary broadly in their specificities) at complex:protease ratios of 1:2500; 1:500; 1:100 and 1:20 (w/w), respectively. Samples were mixed with SDS sample buffer, heated to 95°C for 5 min, and 3 µg of each sample were analyzed by SDS-PAGE and Coomassie staining. "Input" marks the starting material; protease bands are labeled with an asterisk. Note that the complex is remarkably resistant to all but one of the tested proteases tested (Thermolysin), even at very high protease concentrations.

(**C**) **Left:** the Re9F06·RBD·Re5D06 complex was subjected to proteolysis as described in **B**. Samples were analyzed by SDS-PAGE, Coomassie staining and mass spectrometry (for "RBD band 1"/ "RBD band 2", after digestion with trypsin). **Right:** the N-terminal sequence of undigested RBD is shown, with the candidate-P1' sites of Thermolysin highlighted in red. Peptides identified in "RBD band 1" and "RBD band 2", respectively, are indicated.

(**D**) The purified Re9F06·RBD·Re5D06 complex was spiked with Thermolysin (at a 1:500 w/w ratio) and subjected to crystal screening at 20°C. The micrograph shows the crystals obtained in a (scaled-up) 2.5-µl hanging drop of the first hit condition. See "Materials and Methods" for details.

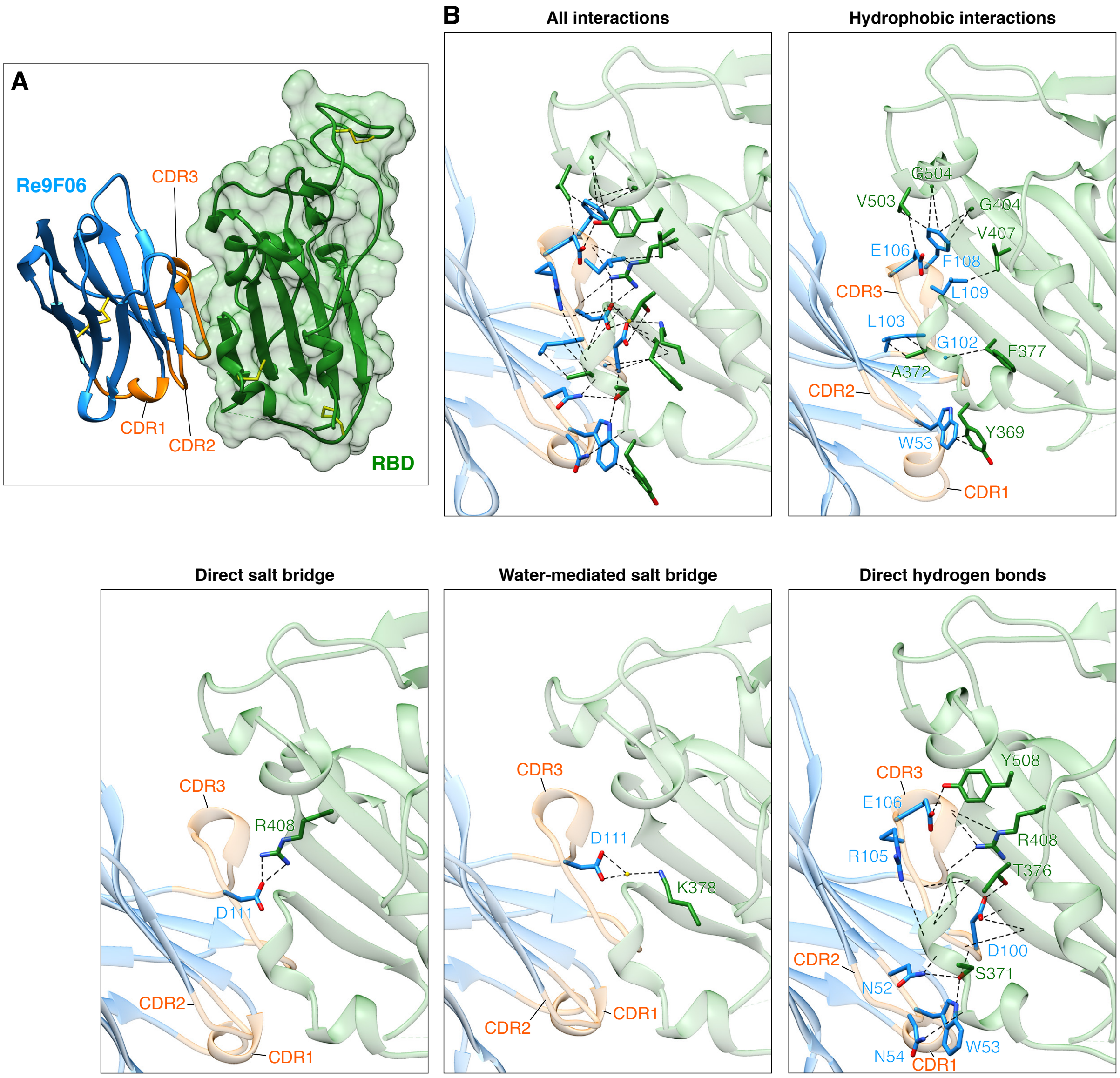

Figure S5 (related to Fig 6)

Molecular details of the Re9F06·RBD interaction, sorted by contact types.

(**A**) Overview of the Re9F06·RBD complex (PDB ID 7OLZ; Re5D06 was omitted for clarity), with the RBD shown as a green ribbon overlayed with its semitransparent surface. Re9F06 is depicted as a ribbon (blue) with the CDR regions highlighted in orange.

(**B**) Details of the Re9F06·RBD interaction interface, either depicting all interactions or interactions sorted by contact type. RBD and Re9F06 are shown as transparent ribbons, colored as in **A**, with interface side chains depicted in green (RBD) or blue (Re9F06). Blue marks nitrogen, oxygen is shown in red, a water molecule is depicted as a yellow sphere. Dashed lines link interacting atoms (distance ≤ 4 Å). Lines pointing onto backbones indicate contacts to carbonyl-carbons or amide groups. Note that the Re9F06·RBD interaction does not show the same degree of shape complementarity as the RBD·Re5D06 complex (Fig 6E, S6).

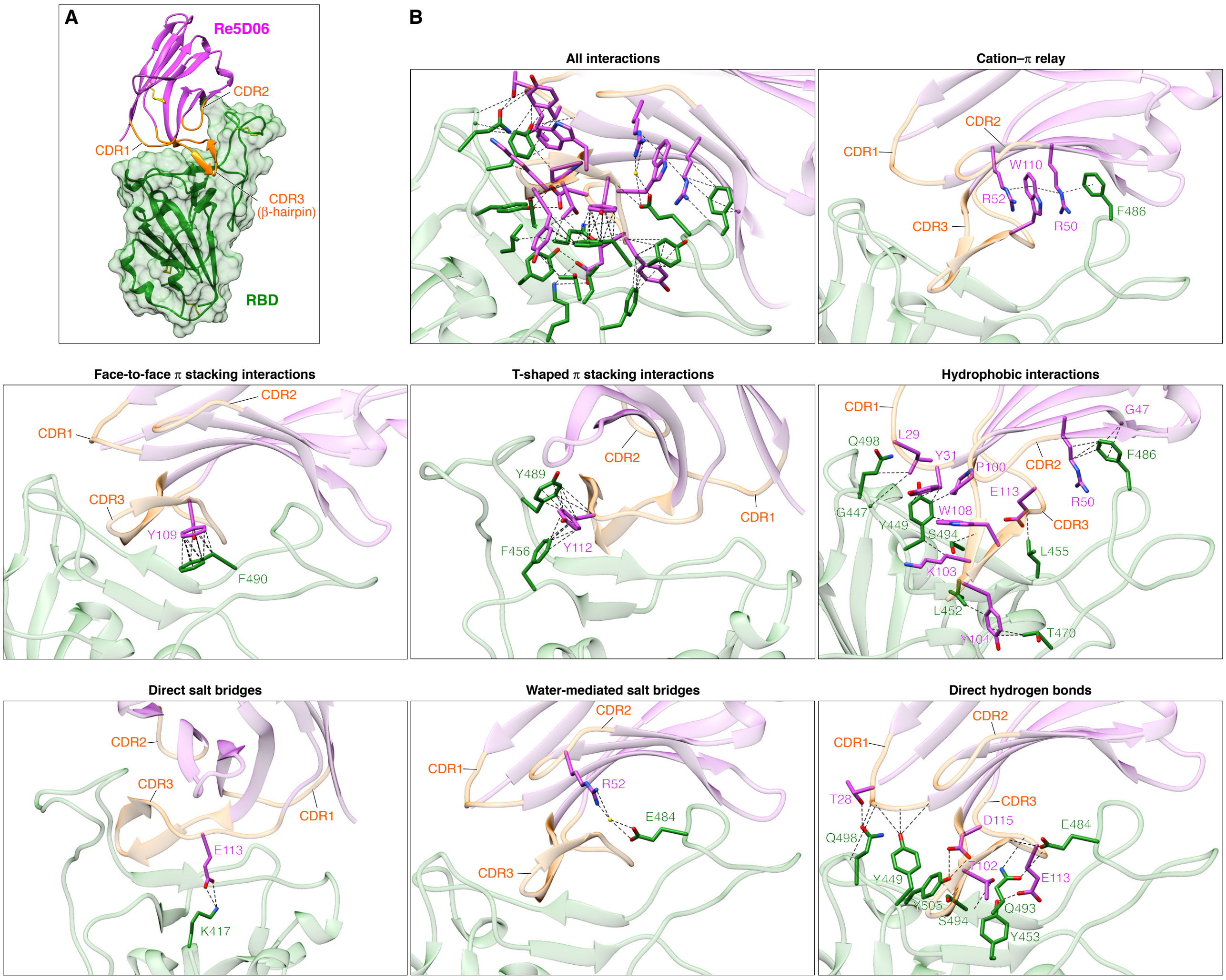

Figure S6 (related to Fig 6)

Molecular details of the RBD·Re5D06 interaction, sorted by contact types.

(**A**) Overview of the RBD·Re5D06 complex (PDB ID 7OLZ; Re9F06 was omitted for clarity), with the RBD shown as a green ribbon overlayed with its semitransparent surface. Re5D06 is depicted as a ribbon (magenta) with the CDR regions highlighted in orange, as in Fig 6E.

(**B**) Details of the RBD·Re5D06 interaction interface, either depicting all interactions (equivalent to Fig 6E, with a few additional contacts) or interactions sorted by contact type. RBD and Re5D06 are shown as transparent ribbons colored as in **A**, with interface side chains depicted in green (RBD) or magenta (Re5D06). Blue marks nitrogen, oxygen is shown in red, a water molecule is depicted as a yellow sphere. Dashed lines link interacting atoms (distance ≤ 4 Å). Lines pointing onto backbones indicate contacts to carbonyl-carbons or amide groups. Note the intramolecular cation-π interactions of Re5D06. These contacts form an array critical for RBD interaction. RBD F486 ("hydrophobic interactions") is also poised to form a cation-π contact with Re5D06 R50.

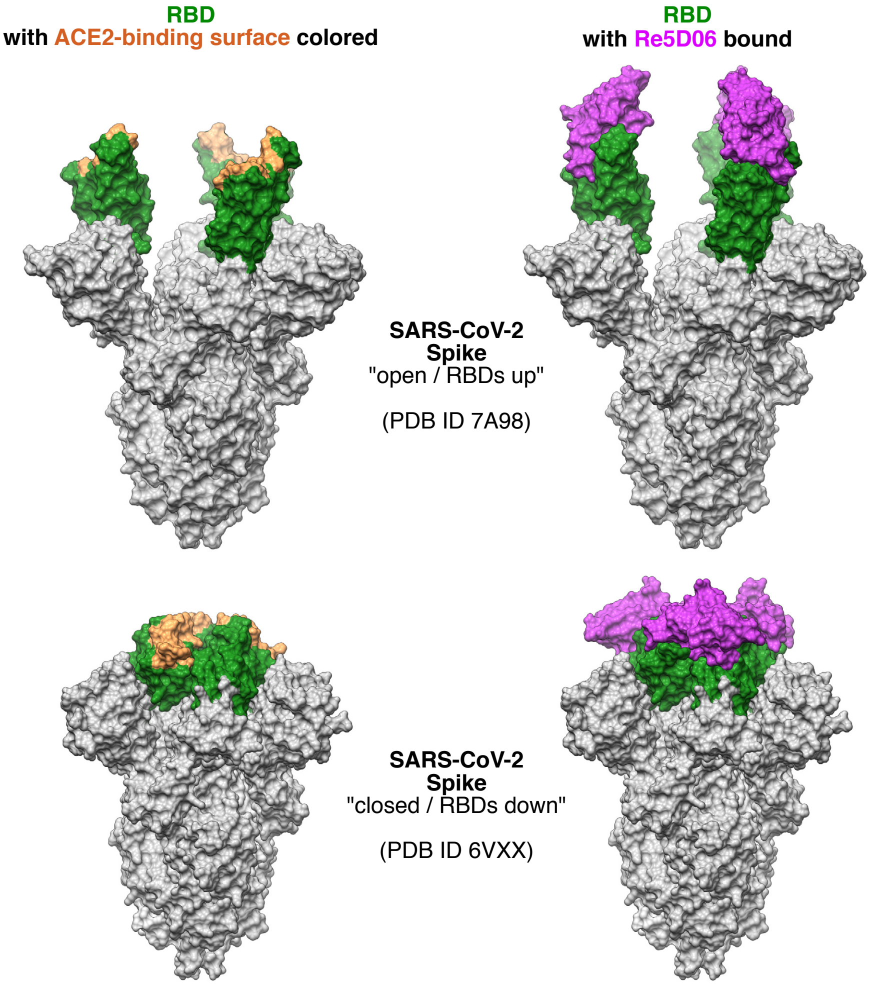

Figure S7 (related to Fig 6C)

Compatibility of VHH Re5D06 with the "open" and "closed" Spike.

Left: surface representation of the SARS-CoV-2 Spike in the "all-RBDs-up" (PDB ID 7A98; Benton *et al*., 2020) or "all-RBDs-down" conformation (PDB ID 6VXX; Walls *et al*., 2020). The RBD is shown in green, with the ACE2-binding surface depicted in brown.

Right: The RBD·Re5D06 complex from Fig 6A was docked into the Spike representations shown on the left, based on the structural alignment of the RBDs. Re5D06 is shown as a surface in magenta. Note that both Spike conformations are compatible with Re5D06 binding.

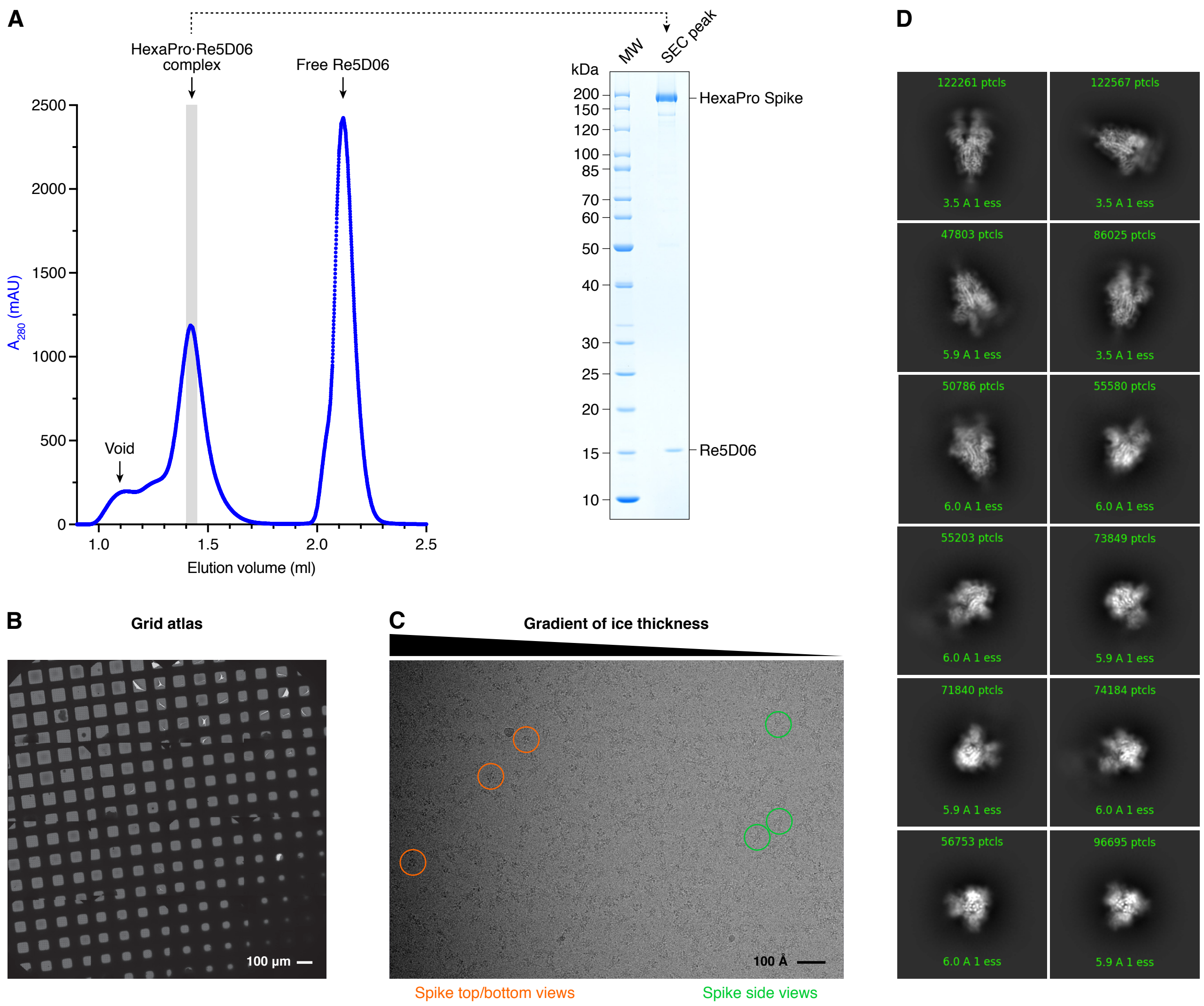

Figure S8 (related to Fig 6C)

Sample and grid preparation for cryo-EM studies.

(**A**) The purified pre-fusion-stabilized SARS-CoV-2 Spike ectodomain trimer ("HexaPro") was mixed with a 9-fold molar excess of VHH Re5D06 and passed over a Superose 6 column. The A_{280} elution profile is shown **(left panel)**, with the peak fraction of the HexaPro·Re5D06 complex highlighted in gray. This fraction was analyzed by SDS-PAGE and Coomassie staining **(right panel)**.

(**B**) The peak eluate fraction was vitrified immediately after elution on Quantifoil UltrAuFoil R2/2 (200 mesh) grids. Grids were screened on a Glacios 200 kV TEM equipped with a Falcon III detector. The micrograph shows the atlas overview of the grid that was later used for data collection on a Titan Krios G2 microscope. Note the gradient of ice thickness across the grid.

(**C**) 15636 movies were collected on a Titan Krios G2 microscope equipped with an energy filter and a K3 detector. We aquired data from holes of a broader range of ice thickness, as illustrated by the shown micrograph: while particle side views dominated in thin ice (green circles), slightly thicker ice also accommodated Spike particles in the top/bottom view orientation.

(**D**) All selected particles were subjected to 2D classification in cryoSPARC. The images show representative 2D class averages. Note that the class averages include side views, edge-on views as well as top/bottom views at high resolution. Particle number, resolution and effective sample size ("ess", with a value of 1 indicating high certainty of the alignment) are shown for each class as reported by cryoSPARC.

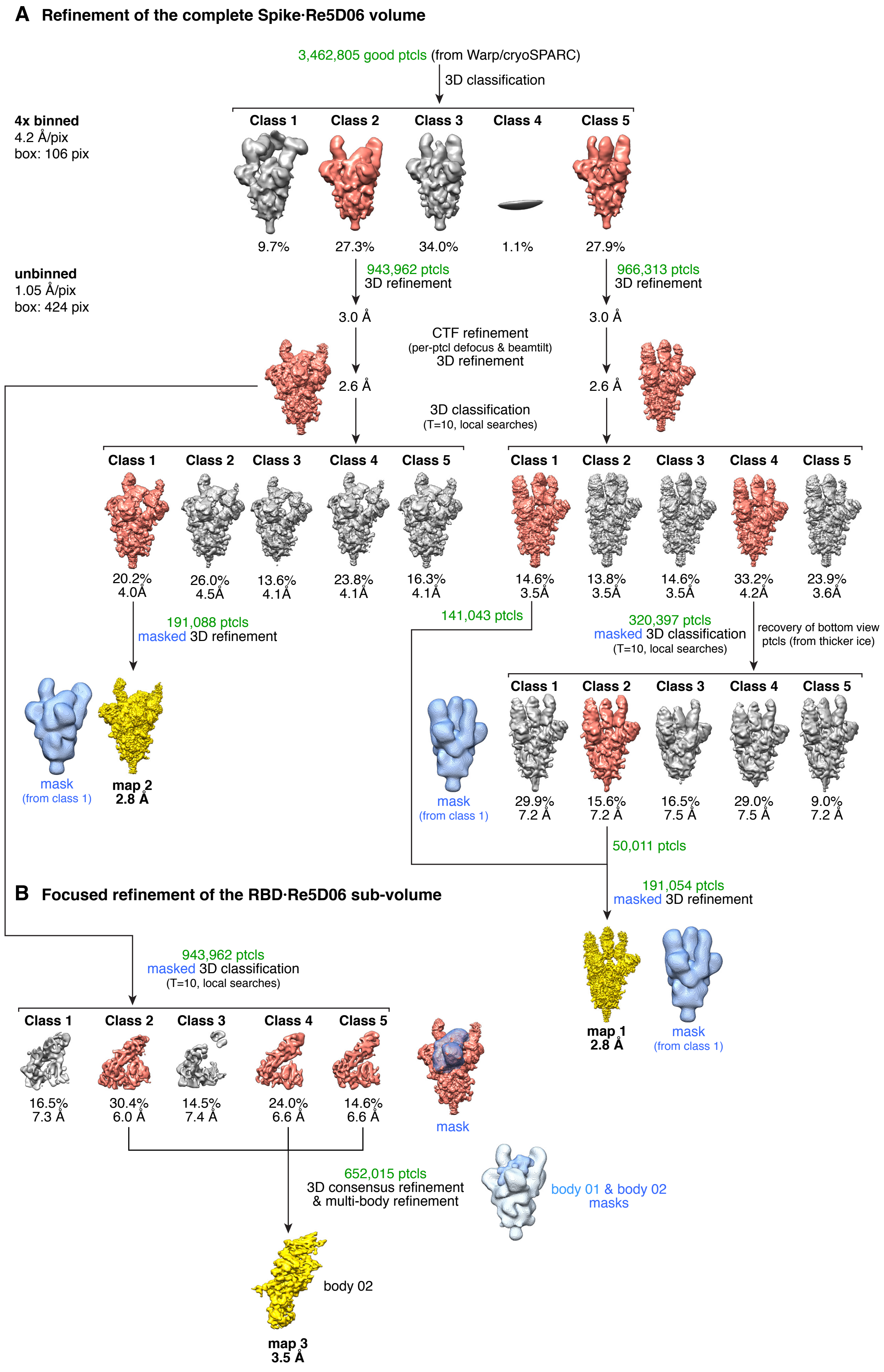
A Refinement of the complete Spike·Re5D06 volume
3,462,805 good ptcls (from Warp/cryoSPARC)
3D classification
4x binned
4.2 Å/pix
box: 106 pix
Class 1
Class 2
Class 3
Class 4
Class 5
9.7%
27.3%
34.0%
1.1%
27.9%
unbinned
1.05 Å/pix
box: 424 pix
943,962 ptcls
3D refinement
966,313 ptcls
3D refinement
3.0 Å
3.0 Å
CTF refinement
(per-ptcl defocus & beamtilt)
3D refinement
2.6 Å
2.6 Å
3D classification
(T=10, local searches)
Class 1
Class 2
Class 3
Class 4
Class 5
20.2%
4.0Å
26.0%
4.5Å
13.6%
4.1Å
23.8%
4.1Å
16.3%
4.1Å
Class 1
Class 2
Class 3
Class 4
Class 5
14.6%
3.5Å
13.8%
3.5Å
14.6%
3.5Å
33.2%
4.2Å
23.9%
3.6Å
191,088 ptcls
masked 3D refinement
141,043 ptcls
320,397 ptcls
masked 3D classification
(T=10, local searches)
recovery of bottom view
ptcls (from thicker ice)
mask
(from class 1)
map 2
2.8 Å
Class 1
Class 2
Class 3
Class 4
Class 5
mask
(from class 1)
29.9%
7.2 Å
15.6%
7.2 Å
16.5%
7.5 Å
29.0%
7.5 Å
9.0%
7.2 Å
50,011 ptcls
191,054 ptcls
masked 3D refinement
map 1
2.8 Å
mask
(from class 1)
B Focused refinement of the RBD·Re5D06 sub-volume
943,962 ptcls
masked 3D classification
(T=10, local searches)
Class 1
Class 2
Class 3
Class 4
Class 5
16.5%
7.3 Å
30.4%
6.0 Å
14.5%
7.4 Å
24.0%
6.6 Å
14.6%
6.6 Å
mask
652,015 ptcls
3D consensus refinement
& multi-body refinement
body 01 & body 02
masks
body 02
map 3
3.5 Å

Figure S9 (previous page; related to Fig 6C)

Image processing workflow for cryo-EM maps 1-3.

The sequence of 3D classification and 3D refinement steps leading to maps 1-3 is shown. All high-resolution image processing was done in RELION (version 3.1.1). Volumes of 3D classes as well as unsharpened maps obtained in intermediate refinements are shown, along with the achieved resolution. The percentage of input particles left in each class is indicated. Classes and 3D refinements highlighted in salmon were used for downstream processing (with absolute particle numbers shown in green); 3D classes shown in gray were discarded. The 3D masks used for focused (masked) 3D classification or masked 3D refinement runs are shown in blue (semi-transparent surface). The final (post-processed) maps are shown in gold.

(**A**) Classification and refinement tree for the complete Spike·Re5D06 volume.

(**B**) Classification and refinement tree for the RBD·Re5D06 sub-volume. Map 3 was obtained by masking both the RBD·Re5D06 volume (body 02 mask) and the remaining volume (body 01 mask) and refining them separately as rigid bodies with partial signal subtraction and centering (as implemented in RELION's multi-body refinement; Nakane *et al*., 2018).

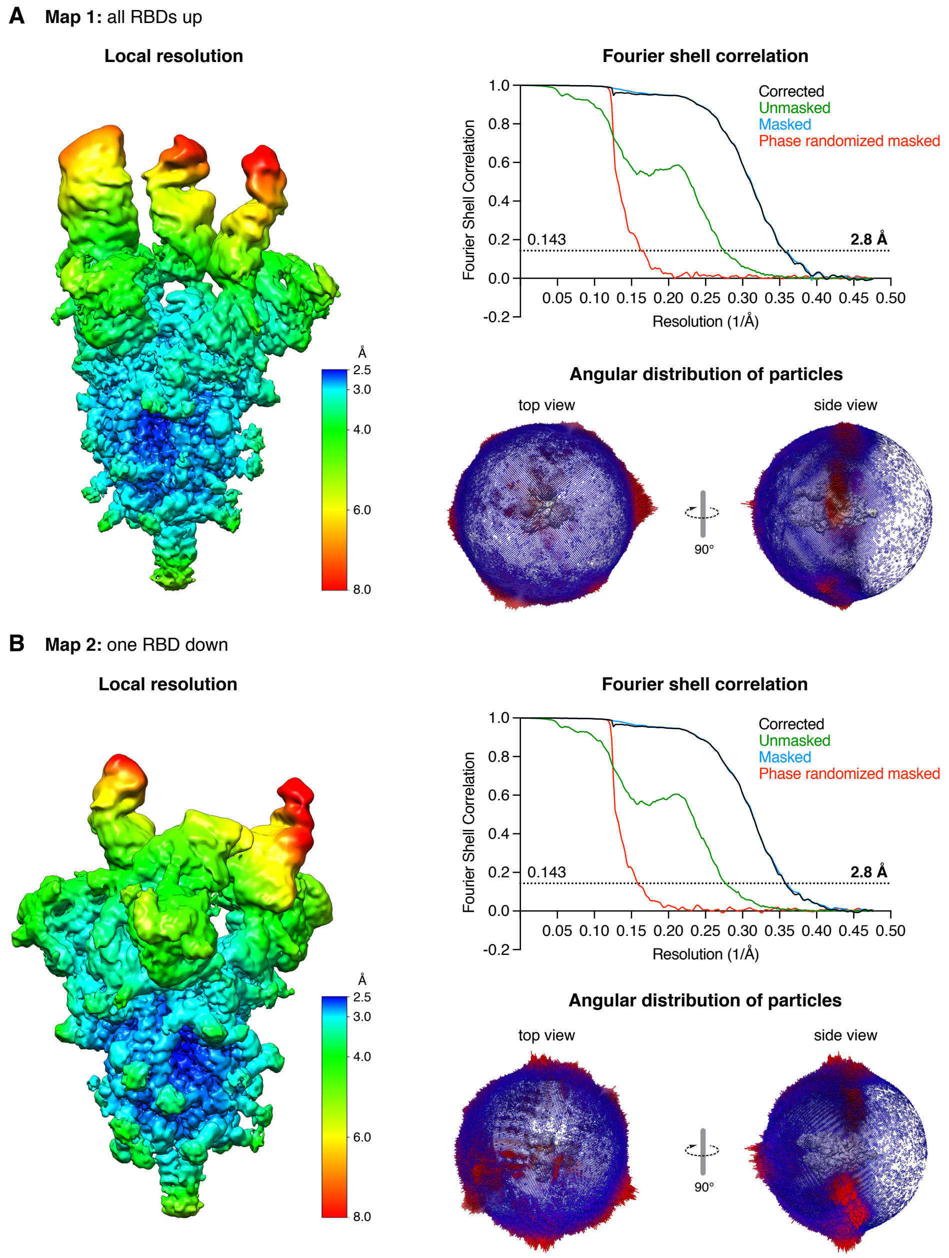

Figure S10 (related to Fig 6C)

Quality of the cryo-EM reconstructions (map 1/EMD-13107 and map 2/EMD-13106).

(**A**) Quality assessment of map 1, which shows all RBDs in an open/up conformation.

Fourier shell correlation (FSC): The plots show the FSC for the indicated half-maps (see color code). The "gold standard" correlation cutoff (FSC=0.143) is highlighted by the dashed line, showing a global resolution of 2.8 Å for this map.

Local Resolution: The locally filtered map was color-coded in relion_postprocess (RELION, version 3.1.1) according to the resolution shown in the scale bar. Even though the local resolution in the periphery of the complex (e.g. the RBD·Re5D06 modules) is lower in this "global" refinement, high-resolution reconstructions could be obtained for these sub-volumes through focused refinements with partial signal subtraction (see Appendix Fig S11 for an example).

Angular distribution: The Euler angle distribution for the particles in the 3D reconstruction is shown for two views (top and side view of the Spike). The height of the bars and the coloring from blue to red represents the number of particles at the given orientation.

(**B**) Quality assessment of map 2, which shows two RBDs in an open/up and one RBD in a closed/down form. See **A** for details.

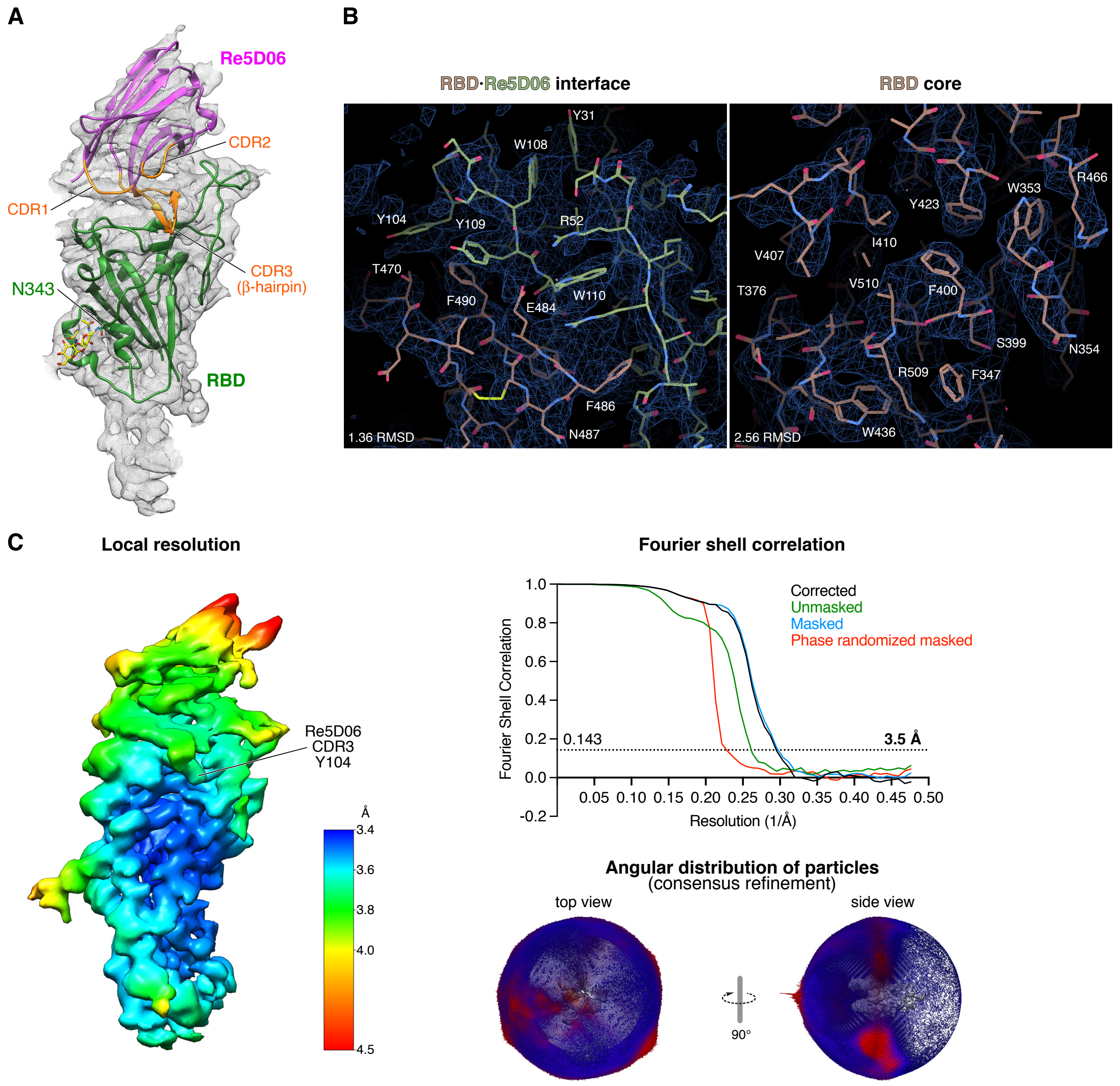

Figure S11 (related to Fig 6C)

Higher-resolution reconstruction of the RBD·Re5D06 sub-volume (map 3/EMD-13105).

The RBD·Re5D06 module of the closed Spike protomer (see map 2) displayed the lowest relative flexibility and was thus chosen for high-resolution multi-body refinement in RELION (see Appendix Fig S9 for details).

(**A**) The crystal structure of the RBD·Re5D06 complex was manually docked into the sharpened cryo-EM density (shown as a gray mesh) and rigid body-refined in Phenix. The model is displayed as in Fig 6E. Note that the cryo-EM map agrees well with the crystal structure. The N-glycosylation at RBD Asn 343 is shown as yellow sticks.

(**B**) Detail views of the RBD·Re5D06 interface (**left**) and the RBD core (**right**) generated in Coot.

(**C**) Fourier shell correlation, local resolution and angular distribution plots for the RBD·Re5D06 sub-volume (map 3). See Appendix Fig S9 and S10A for details.

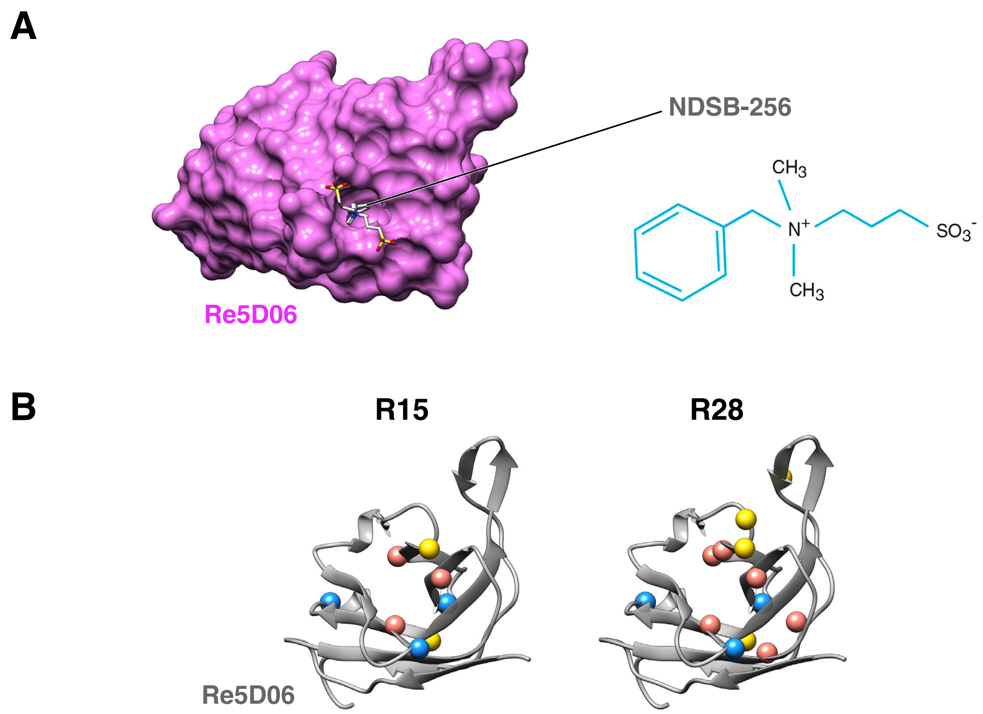

	R15	R28
Optimized hydrophobic core/ packing	I34M<br>S49A<br>V79Y	A24I<br>I27S<br>I34M<br>S49A<br>I51W<br>V79Y
Stabilizing intramolecular salt bridges	Q39E<br>N77D<br>K87E	Q39E<br>N77D<br>K87E
Other changes	S53N<br>V93D	S53N<br>S54N<br>V93D<br>Y109H

Figure S12 (related to Fig 7)

Engineering of hyperthermostable Re5D06 (R) variants.

(**A**) VHH Re5D06 prior to engineering for thermostability. The panel shows a surface representation of Re5D06 (magenta) as in Fig 7D to illustrate a large cavity in the hydrophobic core of the VHH antibody. The electron density map revealed that a molecule of the crystallization additive NDSB-256 (a zwitterionic nondetergent sulfobetaine; Dimethylbenzylammonium Propane Sulfonate) filled this void with its hydrophobic benzyl moiety in the crystal structure of the Re9F06·RBD·Re5D06 complex. Two distinct orientations of the molecule's aliphatic part could be modeled and refined.

(**B**) Point mutations introduced into Re5D06. The table lists the changes for the two Re5D06 variants discussed in this study (R15; R28), sorted by category. The Cα carbons of these altered residues are shown as spheres (color-coded as in the table) on the ribbon representation of Re5D06. The orientation is similar to that of Re5D06 in **A**.

All mutations indicated in red and blue stabilize the nanobody through intramolecular interactions. S53N and S54N (yellow) were found in other members of the Re5D06 sequence class and engage in alternative, possibly more stable intramolecular hydrogen bonding contacts. V93D (yellow) primarily serves to further minimize aggregation of Re5D06, but also improves surface packing. Y109H (yellow, a CDR3 mutation) was introduced to establish charge complementarity to RBD^{E484} while preserving the hydrophobic stacking with RBD^{F490}.

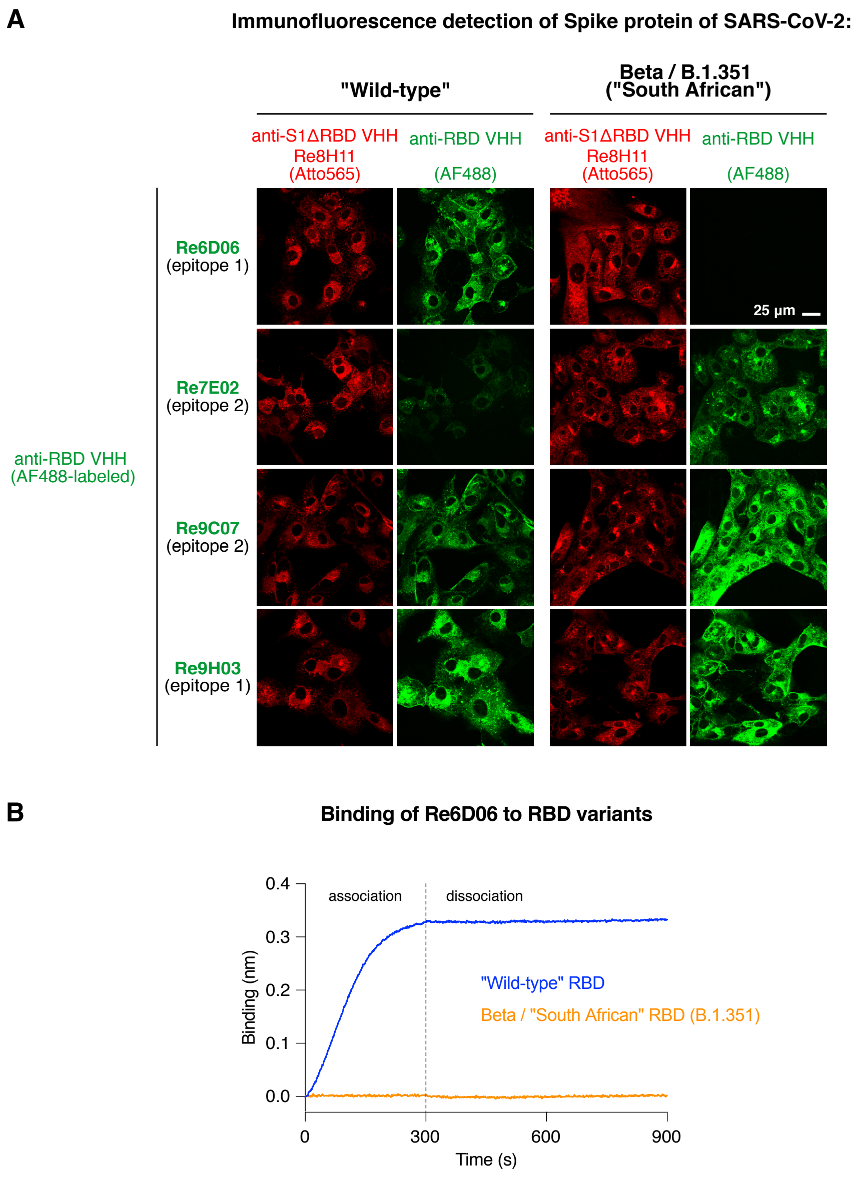

Figure S13 (related to Fig 10)

Nanobody binding to the Spike of the Beta/"South African" SARS-CoV-2 variant.

(**A**) Vero E6 cells were inoculated with the indicated SARS-CoV-2 variants, fixed with paraformaldehyde after two days, stained with 25 nM of the indicated Alexa-Fluor 488 (AF488)-labeled anti-RBD VHH antibodies and 15 nM Re8H11-Atto565 (as an internal control which recognizes Spike epitopes outside the RBD). The cells were imaged by confocal laser scanning microscopy (CLSM). Note that Re6D06 failed to stain B.1.351. Contrary to that, Re7E02 and Re9C07 stained B.1.351 more brightly than wild-type SARS-CoV-2, and Re9H03 was unaffected. This suggests that sets of mutation-sensitive and mutation-tolerant nanobodies can be used to diagnose SARS-CoV-2 variants by straightforward immunostaining or ELISA.

(**B**) BLI experiment to assess binding of Re6D06 (at 20 nM concentration) to the wild-type RBD or the Beta/"South African" B.1.351 RBD variants. Consistent with **A**, Re6D06 rapidly associated with the wild-type RBD, binding it tightly, but it failed to bind the Beta/"South African" RBD mutant.

See Appendix Table S4 for information on naming SARS-CoV-2 variants.

Table S1 (related to Fig 6 and Appendix Fig S4-S6)
Data collection and refinement statistics.
See "Materials and Methods" for further details.

	Complex	
	**Re9F06·RBD·Re5D06**	**Re5D06**
Crystallization	0.1 M MOPS pH 7.5, 2.07 M $(NH_4)_2SO_4$, 0.1 M NDSB-256	100 mM MES pH 6.0, 200 mM $Zn(OAc)_2$, 15% (v/v) ethanol
Cryoprotection	crystallization condition + 20% (w/v) glycerol	crystallization condition
PDB ID	7OLZ	7ON5
**Data collection**		
Space group	$P2_12_12_1$	$P4_12_12$
Cell dimensions		
a, b, c (Å)	55.0 57.0 144.0	44.6 44.6 144.2
α, β, γ (°)	90.0 90.0 90.0	90.0 90.0 90.0
Resolution range (Å)	44.70–1.75 (1.81–1.75)	42.62–1.25 (1.30–1.25)
Unique reflections	45701 (2363)	41182 (3847)
Multiplicity	13.3 (13.0)	23.8 (14.8)
Completeness (%)	85.1 (51.4)	99.4 (95.1)
R_{merge} (%)	9 (153)	5 (40)
R_{pim} (%)	3 (44)	1 (11)
I/σI	17.91 (1.99)	38.44 (7.60)
CC1/2	100 (87.3)	100 (97.3)
**Refinement**		
No. reflections	39591 (2362)	41179 (3846)
R_{work} (%)	17.7 (20.5)	14.1 (19.4)
R_{free} (%)	20.9 (24.6)	16.0 (24.6)
No. atoms		
Protein	3304	1080
Ligands	39	7
Solvent	275	204
B-factors		
Protein	22.82	14.97
Ligands	27.50	24.82
Solvent	30.22	30.60
RMSDs		
Bond lengths (Å)	0.010	0.010
Bond angles (°)	1.06	1.16
MolProbity analysis		
Ramachandran favored (%)	98.07	99.20
Ramachandran outliers (%)	0.00	0.00
Rotamer outliers (%)	1.73	0.00
Clash score	2.62	2.77

Statistics for the highest-resolution shell are shown in parentheses.

Table S2 (related to Fig 6C and Appendix Fig S8-S11)
Cryo-EM data collection and processing statistics.
See "Materials and Methods" for details.

	Map		
	**Map 1**<br>Spike·Re5D06 complex<br>all RBDs up	**Map 2**<br>Spike·Re5D06 complex<br>2 RBDs up, one RBD down	**Map 3**<br>Spike·Re5D06 complex<br>RBD·Re5D06 body
EMDB ID	EMD-13107	EMD-13106	EMD-13105
**Data collection**			
Microscope	Titan Krios G2		
Voltage (kV)	300		
Detector	Gatan K3 Summit<br>(counting, non-super-res.)		
Energy filter slit width	20 eV		
Nominal magnification	81,000		
Pixel size (Å)	1.05		
Exposure time, frames	2 s, 40 frames		
Exposure rate (e^{-}·$pixel^{-1}$·s^{-1})	22.00		
Electron exposure (e^{-}/$Å^{2}$)	39.91		
Defocus range set (µm)	-0.5 to -2.5		
No. of movies collected	15,636		
**Data processing**			
Symmetry imposed	C1		
No. of initial particle images	3,462,805		
No. of final particle images	191,054	191,088	652,015
Average map resolution (Å)*	2.8	2.8	3.5
Map sharpening B factor ($Å^{2}$)	-50	-20	-200
Map resolution range (Å)	2.5 – 10.4	2.5 – 10.6	3.4 – 5.2

(*) FSC = 0.143 (masked)

Table S3
Performance of the lead anti-RBD VHH monomers featured in this study.
Nanobodies are color-coded according to their epitope as in Fig 5A, with epitope 1 binders shown in green and epitope 2 binders displayed in red. For the performance of homo-trimeric VHH fusions see Fig 8. ("n.d.": not determined)

VHH antibody monomer	Tm (°C)	ACE2 competition	IC99+ (pM)	K_D for the wt RBD (pM)
**Re5D06**	65°C	yes	50	2
**(Re5D06) R15**	>95°C	yes	17 – 50	n.d.
**(Re5D06) R28**	>95°C	yes	50	≤1
**Re6B06**	>95°C	yes	50 000	12 000
**Re6D06**	53°C	yes	1700	100
**Re6H06**	>95°C	yes	50	≤1
**Re9B09**	64°C	yes	170	≤1
**Re9H01**	59°C	yes	170	10
**Re9H03**	60°C	yes	170	24
**Re5F10**	>95°C	yes	5000	29
**Re6B07**	58°C	yes	5000	n.d.
**Re6F06**	59°C	yes	5000	n.d.
**Re6H10**	67°C	yes	5000	n.d.
**Re7E02**	59°C	yes	(5000)	n.d.
**Re9C07**	>95°C	no	50 000	50
**Re9F06**	>95°C	yes	17 000-50 000	4 000

IC99+ concentrations shown in parentheses have only been determined by assessing the cytopathic effect (CPE) of the virus.

Table S4

Performance of the lead anti-RBD VHH monomers with the indicated SARS-CoV-2 variants.

The table shows the nanobodies that have been tested with the indicated SARS-CoV-2 variants. Color-coding is according to the recognized epitope as in Fig 5A, with epitope 1 binders shown in green and epitope 2 binders displayed in red. For parameters on homo-trimeric and tandem VHH fusions see Fig 9 and 10. ("wt": wild-type; "SA": South African; "n.b.": no binding)

In addition to the SARS-CoV-2 nomenclature suggested by the World Health Organization (WHO), the PANGO lineage identifier and the obsolete naming of variants by their geographic origin are also provided, because all of these naming conventions have been used in the literature cited in this work.

VHH antibody monomer	Tm (ºC)	ACE2 competition	IC99+ wt (pM)	IC99+ Beta B.1.351 "SA" (pM)	K_D (pM)					
					wt RBD	Alpha B.1.1.7 "UK"	Beta B.1.351 "SA"	Gamma P.1 "Brazilian"	Epsilon B.1.427 B.1.429 "Californian"	Quadruple* Gamma + Epsilon
**Re5D06**	65°C	yes	50		2	20	500	≤ 100	400	
**Re5D06/ R28**	>95°C	yes	50		≤10	≤10	100	≤100	200	
**Re6B06**	>95°C	yes	50 000		12 000	97	n.b.	n.b.	n.b.	
**Re6D06**	53°C	yes	1700		100		n.b.		n.b.	
**Re6H06**	>95°C	yes	50	170	≤1	≤10	≤10	≤10	50	3 000
**Re9B09**	64°C	yes	170	1700	≤1	20	100	100	200	3 000
**Re9H01**	59°C	yes	170		10	400	n.b.	n.b.	200	
**Re9H03**	60°C	yes	170	170	24	50	≤10	≤10	600	150
**Re5F10**	>95°C	yes	5000	1700-5000	29	30	30	30	30	30
**Re9C07**	>95°C	no	50 000		50		50		50	
**Re9F06**	>95°C	yes	17 000-50 000		4 000	200	200	300	200	

* K417T, E484K, N501Y, L452R

Benton DJ, Wrobel AG, Xu P, Roustan C, Martin SR, Rosenthal PB, Skehel JJ, Gamblin SJ (2020) Receptor binding and priming of the spike protein of SARS-CoV-2 for membrane fusion. *Nature*, **588:** 327–330

Nakane T, Kimanius D, Lindahl E, Scheres SH (2018) Characterisation of molecular motions in cryo-EM single-particle data by multi-body refinement in RELION. *Elife*, **7:** e36861

Walls AC, Park YJ, Tortorici MA, Wall A, McGuire AT, Veesler D (2020) Structure, Function, and Antigenicity of the SARS-CoV-2 Spike Glycoprotein. *Cell*, **181:** 281–292.e6
